# Renin angiotensin system blockage by losartan neutralize hypercholesterolemia-induced inflammatory and oxidative injuries

**DOI:** 10.1080/13510002.2020.1763714

**Published:** 2020-05-12

**Authors:** Abdulaziz M.S. AlSaad, Fawaz Alasmari, Hatem M. Abuohashish, Mohamed Mohany, Mohammed M. Ahmed, Salim S. Al-Rejaie

**Affiliations:** a Department of Pharmacology and Toxicology, College of Pharmacy, King Saud University, Riyadh, Saudi Arabia; b Department of Biomedical Dental Sciences, College of Dentistry, Imam Abdulrahman Bin Faisal University, Dammam, Saudi Arabia

**Keywords:** Hypercholesterolemia, losartan, angiotensin, oxidative stress, redox, inflammation, free radicals, antioxidant

## Abstract

**Objectives:** This study explores the protective role of losartan (LT) against oxidative and inflammatory damages in different physiological systems including heart, liver, and kidney tissue in hypercholesterolemic rats.

**Methods:** After induction of hypercholesterolemia by high cholesterol diet for 6 weeks, LT was administered for 4 weeks. In serum, the levels of lipoproteins, aminotransferases, creatine kinases, urea, apoptosis, and inflammatory markers were measured. In cardiac, hepatic, and renal tissues, lipid peroxidation product and GSH as well as antioxidant enzymatic activities were assayed. Finally, histopathological assessment evaluated the structural damage in cardiac, hepatic, and renal tissues.

**Results:** Serum markers of cardiac, hepatic, and renal toxicities including creatine kinases, aminotransferases, and urea were attenuated by LT in hypercholesterolemic animals. Moreover, LT markedly corrected the elevated levels of lipoproteins, apoptosis, and inflammatory biomarkers. Hypercholesterolemia-induced lipid peroxidation, low GSH levels, and diminished activities of antioxidant enzymes were prominently improved in LT treated animals. Histopathological alterations by hypercholesterolemia in heart, liver, and kidney tissues were ameliorated by LT.

**Conclusion:** This study confirmed the pathological enrollment of renin–angiotensin system in hypercholesterolemia-associated metabolic alterations. LT had a significant cardiac, hepatic, and renal protective role against these impairments through down-regulation of oxidative damage, inflammation and necrosis.

## Introduction

High cholesterol is a common metabolic disorder, which is closely related to diabetes and obesity [1–3]. Hypercholesterolemia may deposit fats and triglycerides into the liver, which usually leads to cirrhosis of the liver or even cellular liver cancer [4,5]. Experimental hypercholesterolemia can impair fat metabolism leading to high blood lipids [6]. Furthermore, studies showed that a high cholesterol diet (HCD) could induce hypercholesterolemia even after short term exposure, which is significantly linked to oxidative stress [7]. Furthermore, hypercholesterolemia has multiple serious consequences for different physiological systems. It is considered among the major risk factors for many health problems including ischemic heart disease, fatty liver, and kidney disease. Changing systolic and diastolic blood pressure as well as contractile-induced dysfunction in rodents that are fed on HCD [8] have been reported. Studies showed that cardiac phagocytosis could prevent hypercholesterolemia in rats [9]. Increased cholesterol intake was found to impair the renal function and to provoke kidney damage in rodents [10].

Several molecular pathways have been investigated to identify the mechanisms underlying metabolic disorders associated with high cholesterol. Among these contributing mechanisms, oxidative stress and overproduction of reactive oxygen species (ROS) are commonly documented pathways [11]. Several experimental observations have reported that significantly increased cholesterol loads lead to imbalances in the state of oxidation and reduction within tissues and ROS accumulation. Lipid peroxidation in cellular membranes also appears to be involved as a causal mechanism [11]. Furthermore, studies have revealed the relationship between oxidative stress and inflammation, which was closely related to tissue necrosis and apoptosis under the condition of high cholesterol. Biomarkers of inflammation and elevated programmed DNA damage by HCD were found in rodents [12]. The activation of transcription factors such as nuclear factor kappaB (NF-κB) and the generation of oxidized low-density lipoprotein may explain this association [13].

The renin–angiotensin system (RAS) has one precursor called the angiotensinogen. It is cleaved by kidney-derived renin to form angiotensin (Ang) I, which is subsequently converted to Ang II by angiotensin conversion enzyme (ACE). Ang II is a biologically active peptide in RAS. The main receptor of the pathophysiological and physiological effects of Ang II is the Ang II receptors type 1 (AT1R). Several studies have shown that ACE and AT1R blockers prevent vascular injury caused by high cholesterol in various types [14–17]. Losartan (LT) is an angiotensin II receptor antagonist of the first type used mainly for the treatment of hypertension and diabetic nephropathy. LT intervention to lower hypertension showed that LT was therapeutically superior to atenolol in reducing cardiovascular morbidity and mortality, with a similar decrease in blood pressure [18]. An additional study found that the therapeutic benefits of LT in terms of cardiovascular events were due in part to its ability to decrease low levels of uric acid in the blood [19]. Thus the present study aims to explore the potential protective role of LT on the metabolic and redox status in animals fed on HCD.

## Materials and methods

### Animals and food preparation

Male albino Wistar rats, approximately 70–80 grams (4–6 weeks old), were obtained from the Animal Care Center at King Saud University. After 10 days of acclimatization in standard conditions of temperature, humidity, and day/night cycles, 6 rats were fed with normal cholesterol rat chow (NCRC), while 18 animals were fed HCD for 6 weeks. Water and food were allowed to free access in this completely experiential duration. HCD in pellet form was prepared by adding 1% cholesterol + 0.5% cholic acid with NCRC powder content: protein 20%, fat 4%, fiber 3.5%, ash 6%, and total energy 2850 kcal/kg. The experimental procedures in the present study followed the National Institute of Health guide care policies (NIH 1996). In addition, this study received ethical approval from the Ethical Committee of Pharmacy College, Animal Care Center, King Saud University.

### Experimental design

After 6 weeks of HCD feeding, animals were randomly divided into four groups (*n* = 6) as follows: Group 1, Control group of rats fed with rat chow and treated with vehicle. Group 2, HCD fed rats were treated with vehicle. Group 3, HCD fed rats were treated with LT (10 mg/kg/day, orally) for 4 weeks and Group 4, HCD fed rats were treated with LT (20 mg/kg/day, orally) for 4 weeks. During the LT supplementation, HCD feeding was continued until the end of experiment. Weekly animals’ body weight and general health conditions were carefully monitored during the whole period. Under light anesthesia, blood samples were collected through cardiac puncture and centrifuged at 1800 RCF for 10 min. Serum samples were stored after suppuration at −20°C until analysis. Then, animals were decapitated and dissected to collect heart, liver, and kidney. Tissues were immediately dipped into liquid nitrogen for 1 min, and then stored at −80°C until analysis. A cross section of heart, liver, and kidney were preserved in 10% formaldehyde for histopathological evaluations.

### Serum analysis

Total cholesterol (TC), triglycerides (TG), low-density lipoprotein-cholesterol (LDL), high-density lipoprotein-cholesterol (HDL), creatine kinase-B (CK-B), creatine kinase-MB (CK-MB), alanine aminotransferase (ALT), aspartate aminotransferase (AST), creatinine, and urea levels were estimated by using commercially available diagnostic kits (Human, Wiesbaden, Germany). Inflammatory and apoptotic biomarkers including tumor necrosis factor-alpha (TNF-α), interleukin-1beta (IL-1β), interleukin-6 (IL-6), prostaglandin E-2 (PGE-2), caspase 3 and nitric oxide (NO) levels were estimated by using ELISA kits for rats (R&D systems Inc., USA).

### Tissue analysis

In homogenates of heart, liver, and kidney tissues, thiobarbituric acid reactive substances (TBARS) and glutathione (GSH) levels were measured by using commercially available kits (Cayman Chemical Co., USA). In Post-mitochondria supernatants of heart, liver, and kidney, enzymatic activities of superoxide dismutase (SOD), catalase (CAT), glutathione peroxidase (GPx) and glutathione-S-transferase (GST) were determined using commercially available assay kits (R&D systems Inc., USA).

### Histopathological procedures

Across sectional portions of heart, liver, and kidney tissues from each group were preserved in 10% buffered formalin. After embedding in paraffin blocks, samples were sectioned by rotary microtome to 5 µm sections. These sections were stained with hematoxylin and eosin (H&E) stain and examined for histopathological changes in a blinded manner. The extent of cardiomyocytes damage and hemorrhage, hepatic inflammation and ballooning degeneration and glomerular damage were histologically assessed and scored according to Ma et al. [20]. In brief, cardiomyocytes damage was scored as follows: (0) normal cardiomyocytes with homogenous cytoplasm, (1) less than 2 areas of hemorrhage between muscle fibers, (2) between 2 and 4 areas of hemorrhage, and (3) more than 4 areas of hemorrhage. Hepatic inflammation and ballooning degeneration was scored as follows: (0) no inflammatory foci or ballooned cells, (1) less than 2 inflammatory foci with few ballooned cells, (2) between 2 and 4 inflammatory foci with ballooned cells, and (3) more than 4 inflammatory foci with high number of ballooned cells. The extent of glomerular damage was scored as follow (0) normal, (1) changes <25% of cortical area, (2) changes 25–50% of cortical area, (3) changes 50–75% of cortical area, and (4) changes >75% of cortical area. The mean score of each group was calculated and determined on five randomly chosen fields.

### Statistical analysis

Data were expressed as mean ± standard error of the mean (SEM) and analyzed using one-way analysis of variance (ANOVA) followed by the Student–Newman–Keuls multiple comparisons test (*n* = 6). Differences between groups were considered statistically significant when *P* ≤ 0.05. All statistical tests were conducted using GraphPad Prism software, version 5 (GraphPad Software, Inc., La Jolla, CA, USA).

## Results

In HCD fed rats, levels of TC, TG, and LDL were increased significantly (*P* < 0.001) compared to control animals. Treatment with LT (10 and 20 mg/kg/day) resulted in a significant reduction in the serum levels of TC (*P* < 0.001), TG (*P* < 0.01) and LDL (*P* < 0.05) compared to the HCD group. However, HDL levels did not markedly alter in HCD group when compared to controls. In HCD rats, serum activities of CK-B and CK-MB were increased (*P* < 0.001), while LT (10 and 20 mg/kg/day) treatment revealed significantly (*P* < 0.01) less CK-B and CK-MB activity compared to the HCD fed group. AST and ALT activities were increased significantly (*P* < 0.01) in rats fed with HCD, with lower levels of activity in LT treated animals (*P* < 0.01) as compared to the HCD group. Creatinine and urea levels were significantly (*P* < 0.001) increased in HCD fed animals, which were markedly reduced, relative to HCD fed animals (*P* < 0.05), in LT treated animals (Table 1).

**Table 1.** Effects of LT on hypercholesterolemia-induced changes in serum biochemistry.

Parameters	Control	HCD	LT(10)	LT(20)
TC (mg/dl)	47.95 ± 7.42	112.94 ± 28.51***^a^	69.84 ± 22.45***^b^	60 ± 10***^b^
TG (mg/dl)	21.03 ± 9.24	59.05 ± 12.24***^a^	43.11 ± 6.67**^b^	38.477 ± 6.79**^b^
LDL (mg/dl)	37.79 ± 12.43	55.24 ± 10.59*^a^	37.94 ± 13.24**^b^	37.95 ± 6.2***^b^
HDL (mg/dl)	37.8 ± 6.23	32.56 ± 6.85	33.33 ± 2.54	34.37 ± 2.82
CK-B (U/L)	10.26 ± 165	22.08 ± 3.74***^a^	14.21 ± 4.45***^b^	12.32 ± 2.89***^b^
CK-MB (U/L)	20.54 ± 3.3	44.19 ± 11.48***^a^	26.25 ± 6.99***^b^	23.33 ± 8.3***^b^
ALT (U/L)	17.65 ± 2.16***^a^	36.93 ± 5.96	28.61 ± 5.10**^b^	22.12 ± 4.90***^b^
AST (U/L)	36.67 ± 6.94	54.17 ± 4.67**^a^	41.24 ± 7.50**^b^	37.40 ± 8.75^b^
Creatinine (mg/dl)	2.06 ± 0.67	6.18 ± 2.00***^a^	4.12 ± 0.92*^b^	3.95 ± 1.23*^b^
Urea (mg/dl)	19.87 ± 3.93	59.60 ± 11.79***^a^	39.19 ± 10.10**^b^	32.44 ± 10.59***^b^

Data were expressed as mean ± SEM (*n* = 6) and analyzed using one-way ANOVA followed by Student Newman–Keuls as post hoc test. ^a^Control vs. HCD group; ^b^HCD vs. LT(10) or LT(20). *P* values consider significant when *P < 0.05, **P < 0.01 and ***P < 0.001. ***Abbreviations:*** Total cholesterol (TC); triglycerides (TG); low-density lipoprotein-cholesterol (LDL); high-density lipoprotein-cholesterol (HDL); creatine kinase-B (CK-B); creatine kinase-MB (CK-MB); alanine aminotransferase (ALT); aspartate aminotransferase (AST).

**Figure 1.**
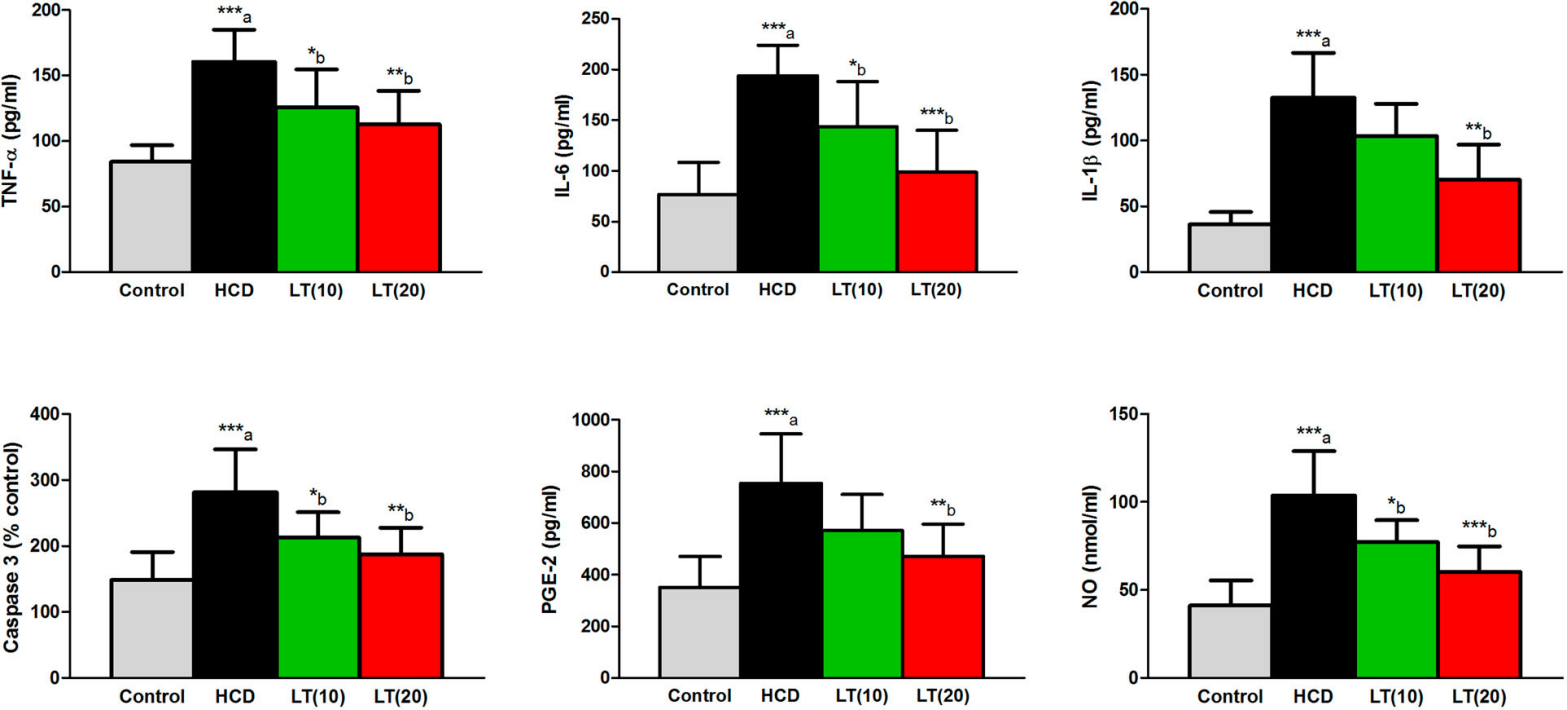
Effect of LT on hypercholesterolemia-induced changes in serum inflammatory biomarkers including TNF-α, IL-6, and IL-1β levels along with serum PGE-2, caspase 3, and NO levels. Data were expressed as mean ± SEM (*n* = 6) and analyzed using one-way ANOVA followed by Student Newman–Keuls as post hoc test. (a) Control vs. HCD group; (b) HCD vs. LT(10) or LT(20). *P* values consider significant when *P < 0.05, **P < 0.01 and ***P < 0.001. Abbreviations: tumor necrosis factor-alpha (TNF-α); interleukin-6 (IL-6); interleukin-1beta (IL-1β); prostaglandin E-2 (PGE-2); nitric oxide (NO).

Serum levels of IL-6, IL-1β, and TNF-α were significantly (*P* < 0.001) increased in HCD fed animals compared to controls. LT treatment to HCD fed rats markedly reduced the levels of IL-6 (*P* < 0.05 and *P* < 0.001; respectively), IL-1β (*P* < 0.01) and TNF-α (*P* < 0.01) compared to HCD treatment alone. NO and caspase-3 activities were significantly (*P* < 0.001) increased in HCD fed rats and these levels were markedly (*P* < 0.05 and *P* < 0.01) reduced in LT (10 and 20 mg/kg/day) treated groups. Serum PGE2 levels were significantly (*P* < 0.001) increased in HCD fed rats. LT (20 mg/kg/day) treatment markedly (*P* < 0.05) reduced PGE2 levels (Figure 1) relative to HCD treatment alone.

**Figure 2.**
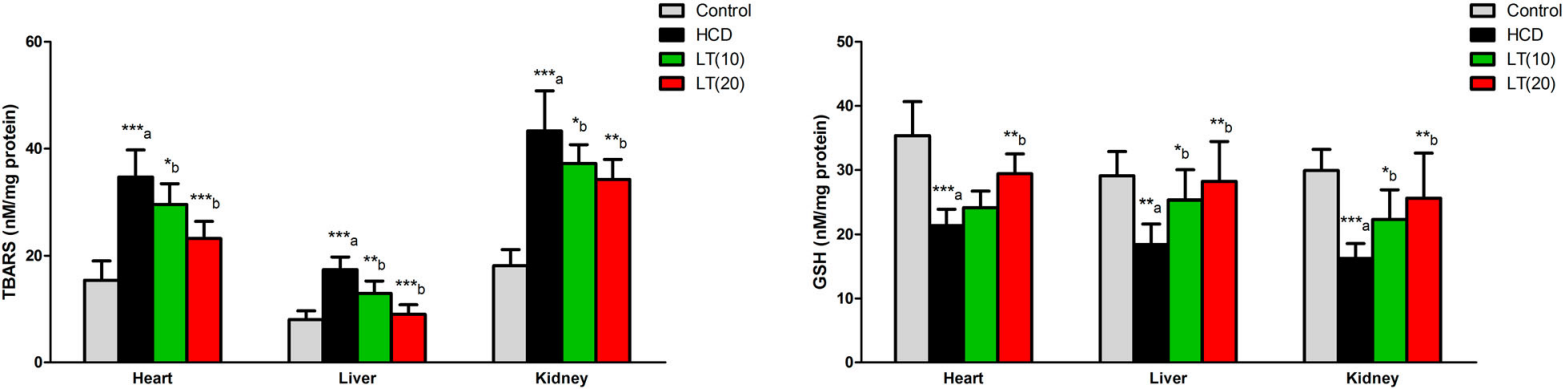
Effect of LT on hypercholesterolemia-induced changes in TBARS and GSH levels in cardiac, hepatic, and renal cells. Data were expressed as mean ± SEM (*n* = 6) and analyzed using one-way ANOVA followed by Student Newman–Keuls as post hoc test. (a) Control vs. HCD group; (b) HCD vs. LT(10) or LT(20). *P* values consider significant when *P < 0.05, **P < 0.01 and ***P < 0.001. Abbreviations: thiobarbituric acid reactive substances (TBARS); glutathione (GSH).

In cardiac, hepatic, and renal cells, TBARS levels were significantly (*P* < 0.001) increased in HCD fed rats. LT treatment (10 and 20 mg/kg/day) markedly reduced the TBARS levels in cardiac (*P* < 0.05 and *P* < 0.001; respectively), hepatic (*P* < 0.01 and *P* < 0.001; respectively) and renal (*P* < 0.05 and *P* < 0.01; respectively) tissues as compared to HCD supplemented rats. HCD fed rats showed significantly decreased GSH levels in heart, liver, and kidney tissues (*P* < 0.001, *P* < 0.01 and *P* < 0.001; respectively) compared to controls. The GSH levels were increased significantly by LT treatment in cardiac (*P* < 0.01), hepatic (*P* < 0.05 and *P* < 0.01; respectively) and renal (*P* < 0.05 and *P* < 0.01; respectively) tissues as compared to HCD supplemented rats (Figure 2).

**Figure 3.**
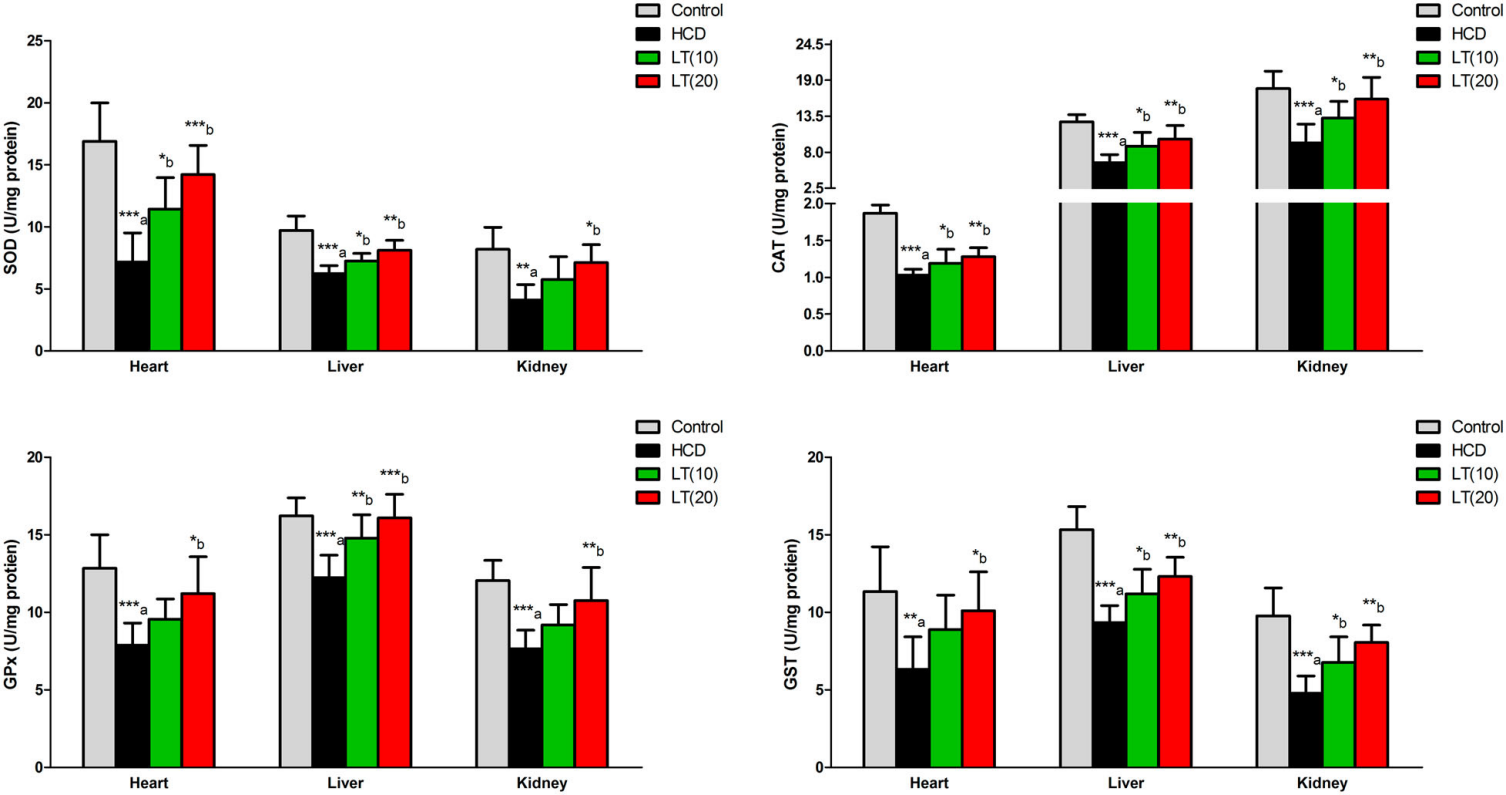
Effect of LT on hypercholesterolemia-induced changes in antioxidant enzymes activities including SOD, CAT, GPx, and GST in cardiac, hepatic, and renal cells. Data were expressed as mean ± SEM (*n* = 6) and analyzed using one-way ANOVA followed by Student Newman–Keuls as post hoc test. a Control vs. HCD group; (b) HCD vs. LT(10) or LT(20). *P* values consider significant when *P < 0.05, **P < 0.01 and ***P < 0.001. Abbreviations: superoxide dismutase (SOD); catalase (CAT); glutathione peroxidase (GPx); glutathione-S-transferase (GST).

The cardiac, hepatic, and renal SOD enzymatic activities were found significantly reduced (*P* < 0.001, *P* < 0.001, and *P* < 0.01; respectively) in HCD fed rats. Both the doses of LT treatment markedly enhanced SOD activities in cardiac (*P* < 0.05 and *P* < 0.001; respectively), hepatic (*P* < 0.05 and *P* < 0.01; respectively) and renal (*P* < 0.05) tissues compared to HCD group**.** CAT enzymatic activities in cardiac, hepatic, and renal cells were significantly reduced (*P* < 0.001) in HCD fed rats. LT treatment markedly enhanced CAT activities in cardiac (*P* < 0.05 and *P* < 0.01; respectively), hepatic (*P* < 0.05 and *P* < 0.01; respectively) and renal (*P* < 0.05 and *P* < 0.01; respectively) tissues as compared to HCD group**.** In heart, liver, and kidney tissues, GPx activities were significantly (*P* < 0.001) decreased in HCD fed rats. HCD fed rats also treated with LT (10 and 20 mg/kg/day) had markedly enhanced GPx enzymatic activities in cardiac (*P* < 0.05), hepatic (*P* < 0.01 and *P* < 0.001; respectively) and renal (*P* < 0.01) tissues as compared to HCD treatment alone. GST enzymatic activities in cardiac, hepatic, and renal tissues were significantly diminished (*P* < 0.01, *P* < 0.001 and *P* < 0.001; respectively) in the HCD group. Treatment of the HCD supplemented animals with LT (10 and 20 mg/kg/day) markedly enhanced GST activities in cardiac (*P* < 0.05), hepatic (*P* < 0.05 and *P* < 0.01; respectively) and renal (*P* < 0.05 and *P* < 0.01; respectively) tissues as compared to HCD treatment alone (Figure 3).

**Figure 4.**
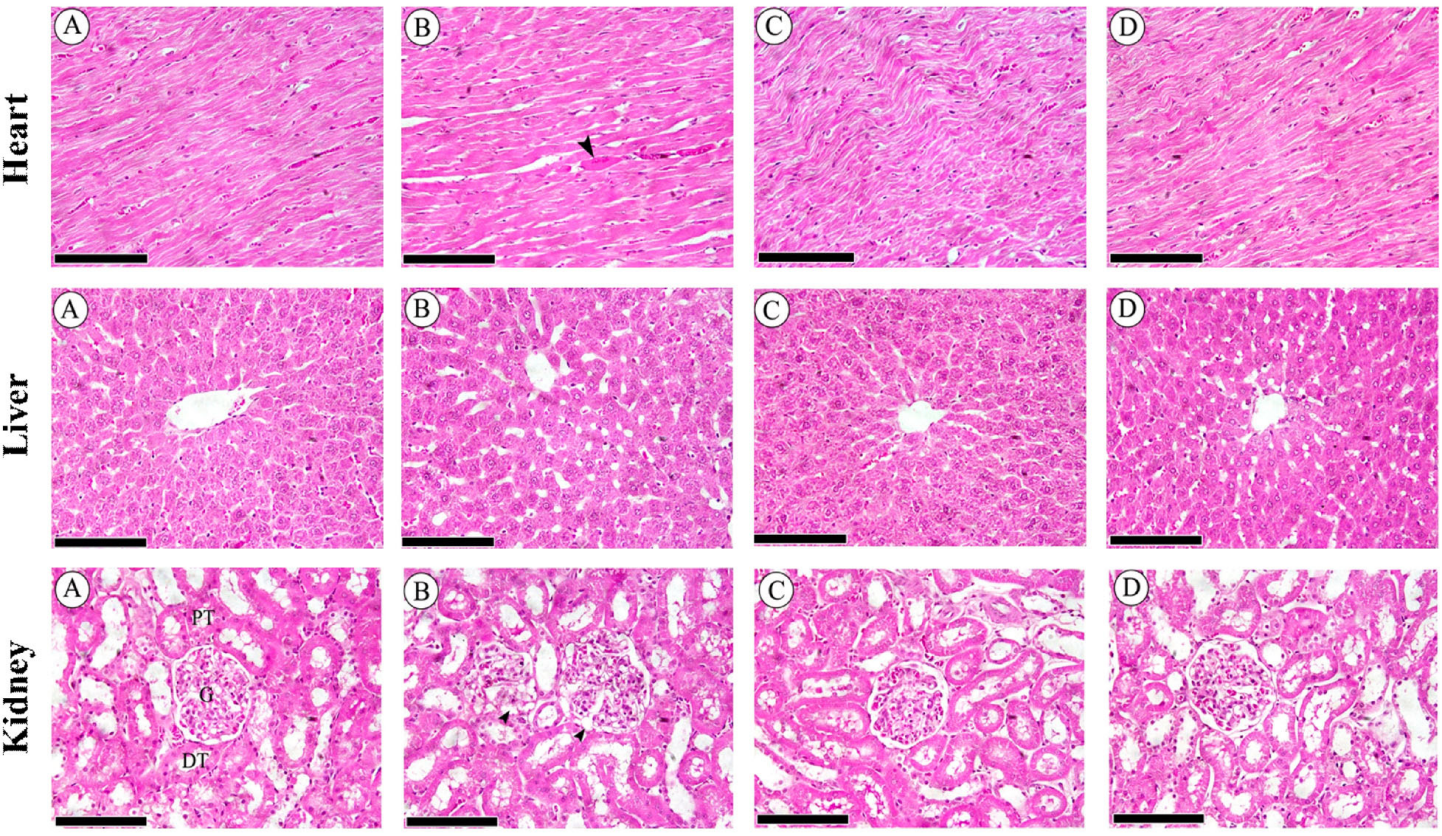
Sections of heart tissues (A–D) showing (A) the control group with normal appearance of myocardial cells, (B) severe damage with hemorrhage between muscle fibers (arrow) in HCD group, (C) less injury and normal myocardial cell morphology with oval-elongate nucleus centrally and homogeneous cytoplasm in LT(10) group, (D) almost normal cardiomyocytes looking in LT(20) group. Sections of hepatic tissues (A–D) showing (A) normal architecture of hepatocytes and central vein in control group, (B) steatosis and infiltration of inflammatory cells in HCD group, (C) incomplete regenerating hepatocytes around the central vein in LT(10) group, (D) complete recovery in hepatocytes with binuclear cells in LT(20) group. Sections of renal cortex (A–D) showing (A) normal appearance of the proximal convoluted tubules [PT], distal convoluted tubules [DT], Bowman's capsule and glomerulus [G] in control group, (B) dilatation in glomerular capillaries (head arrow), increase in glomerular tuft size and expansion in Bowman's space in HCD group, (C) partial improvement in glomeruli and renal tubules in LT(10) group, (D) complete retrieval of glomeruli and renal tubules in LT(20) group. Scale bar = 50 µm.

The semi-quantitative histological assessment of cardiac, hepatic, and renal sections was showed in Table 2. Histological changes in cross sections of heart tissues from the control group showed normal appearance of myocardial cells with oval elongated nuclei and homogenous cytoplasm. Rats fed HCD showed marked damage with hemorrhage between muscle fibers. Rats treated with LT (10 mg/kg/day) had less injury and normal morphology of the myocardial cells along with homogeneous cytoplasm and oval-elongate nucleus. The higher dose of LT (20 mg/kg/day) showed significant less change in cardiomyocytes (Figure 4). Liver sections from a control rats revealed normal architecture of hepatocytes, while liver sections from the HCD fed group showed steatosis and inflammatory infiltration. In the LT treated group (10 mg/kg/day), there was incomplete regenerating hepatocytes around the central vein. Liver sections from LT (20 mg/kg/day) treated rats showed complete recovery in hepatocytes with binuclear cells (Figure 4). Representative histology images of the renal cortex of control rats revealed normal appearance of the proximal and distal convoluted tubules as well as Bowman's capsule. Rats fed on HCD showed dilated glomerular capillaries, and expanded glomerular tuft and Bowman's space. Treatment of HCD animals with LT in two doses (10 and 20 mg/kg/day) appeared to dose dependently abrogate negative histological changes in glomeruli and renal tubules (Figure 4).

**Table 2.** Quantitative assessment of LT effects on hypercholesterolemia-induced changes in histological features of heart, liver, and kidney tissues.

Tissue	Control	HCD	LT(10)	LT(20)
Heart	0.12 ± 0.070	1.05 ± 0.213**^a^	0.60 ± 0.086	0.41 ± 0.070*^b^
Liver	0.20 ± 0.030	2.00 ± 0.200**^a^	1.80 ± 0.170	1.20 ± 0.150*^b^
Kidney	0.53 ± 0.020	1.40 ± 0.180*^a^	1.00 ± 0.160	0.57 ± 0.210*^b^

Data were expressed as mean ± SEM (*n* = 6) and analyzed using one-way ANOVA followed by Student Newman–Keuls as post hoc test. ^a^Control vs. HCD group; ^b^HCD vs. LT(10) or LT(20). *P* values consider significant when *P < 0.05, **P < 0.01 and ***P < 0.001.

## Discussion

Hypercholesterolemia due to chronic consumption of HCD has detrimental effects on multiple physiological systems, which may lead to altered redox and metabolic status. In the present study, experimentally induced hypercholesterolemia following 6 weeks of HCD feeding in rats resulted in impaired cardiac, hepatic, and renal structure and function. These effects were associated with elevated oxidative stress and inflammatory activity. The pathological role of RAS during hypercholesterolemia has been documented. In the current study, blockage of terminal step of RAS by LT markedly improved the altered cardiac, hepatic, and renal architecture and physiology *via* reducing local and systemic oxidative stress and inflammatory cytokines.

In the present study, we determined several biomarker of oxidative stress in different biological tissue. Hypercholesterolemia-induced oxidative stress, which triggered lipid peroxidation and reduced GSH levels. Furthermore, the activities of antioxidant enzymes, which prevent ROS oxidative destructions, were diminished in HCD fed animals. At the early stage of oxidative stress, the expressions and activities of antioxidant enzymes are increased, which is considered a defensive mechanism to protect cellular functions [21]. Reposts also show that free radicals initially induce antioxidant genes [21]. However, the activities of these antioxidant enzymes are diminished upon prolonged exposure to oxidative stress. This could be explained by the concept of ‘defense exhaust’, where cells are not able to produce these enzymes in response to high and incessant free radicals attack. In addition, antioxidant enzymes themselves are vulnerable to degradation by free radicals [21].

Hypercholesterolemia is known to trigger monocytes formation of systemic inflammatory cytokines [22]. These inflammatory mediators play critical function in the pathogenesis of multiple metabolic syndromes, which made them a promising panel for the assessment of cardiovascular and metabolic disorders. In the present study, HCD feeding significantly increased serum levels of IL-6, IL-1β, TNF-α, and PGE2, which indicates an advanced degree of systemic inflammation. These results are in agreement with Chan et al., where experimental hypercholesterolemia was associated with reduced antioxidant capacity and systemic inflammation [23]. Furthermore, HCD fed animals showed a marked increase in the serum level of caspase-3 and NO. These biological molecules are markers for cellular damage and apoptosis. Studies have showed that hypercholesterolemia could noticeably trigger apoptosis [24] and formation of NO [25]. The elevated systemic inflammation and apoptosis in hypercholesterolemic rats might be attributed to combined oxidative and nitrosative cellular injuries, which heightened vascular permeability as well as leucocyte trafficking [26]. RAS has also been linked to the pathophysiology hypercholesterolemia-induced systemic inflammation. Ang II was found to enhance monocytes migration and pro-inflammatory cytokine levels throughout hypercholesterolemia [27]. In addition, Ang II is known to promote and regulate the formation of ROS and NO [28]. Contestant with this, inhibition of the eventual step in the Ang II cascade through blocking AT1R might show a considerable reduction in systemic inflammatory mediators and caspase-3, which was reported in LT treated animals in the current study.

Elevated levels of cholesterol in the blood can exert direct harmful effects on the myocardium. Numerous clinical and preclinical studies have explored the deleterious effects of hypercholesterolemia on cardiac performance and contractile dysfunction [29]. In the present study, serum levels of TC, TG, and LDL were markedly increased in HCD fed animals, while HDL levels were reduced, which indicates systemic hypercholesterolemia. Moreover, high cholesterol levels evoked cardiomyocytes destruction, which explains the elevation of serum levels of CK-B and CK-MB. In addition, histological analysis revealed hypercholesterolemia-induced alterations among the architecture of cardiac muscle fibers. This is in agreement with other studies, where cholesterol lowering therapy ameliorated cardiac failure in hypercholesterolemic rodents [30]. Hypercholesterolemia-induced cardiac damage in the current study may also attribute to down-regulation of antioxidant enzymes and GSH and provoked lipid peroxidation. Other studies showed that free radicals scavenging by antioxidant compounds demonstrated enhanced myocardial necrosis, properties, and redox status in HCD rats [12]. Interestingly, modulation of AT1R was found to reduce hypercholesterolemia-associated impairments associated with myocardial ischemic-reperfusion [31]. Furthermore, RAS inhibition resulted in improved serum lipids and energy consumption in cardiac tissue in HCD fed rabbits [32]. Similarly, we found that LT can preserve cardiac muscle from oxidative injuries *via* improving GSH levels and antioxidant enzyme activates and reduce lipid peroxidation. The cardio-protective effects of LT were further confirmed by the histological analysis.

Liver functions and hepatic cellular structure are known to be sensitive to changes in serum cholesterol levels. Hypercholesterolemia was found in multiple studies to impair liver function test and to alter the histological structure of the liver [33,34]. In the current study, serum levels of ALT and AST were elevated in HCD rats, while histological features of hepatic slides showed steatosis and inflammation. HCD induced-oxidative stress and lipid peroxidation may explain the reported damage and necrosis. In one study, hypercholesterolemic animals demonstrated significant hepatic lipid peroxidation and reduced SOD and CAT functions [35]. Modulation of AT1R by LT markedly protected against hypercholesterolemia-induced alterations in liver functions and histology structure. LT is known to possess hepato-protective effects in several conditions, where oxidative stress is deemed to play a pathological role. For instance, El-Demerdash et al found that LT attenuates carbon tetrachloride-induced liver fibrosis and oxidative stress [36]. Moreover, LT markedly lowered diabetic associated oxidative hepatic damages in rats in another study [37]. Likewise, we reported that LT treatment corrects the altered redox status and oxidative injuries in liver tissues of hypercholesteremic animals.

Kidney tissues and renal functions were also altered by hypercholesterolemia in the present study. Feeding of the animals with HCD markedly elevated the levels of creatinine and urea, which are markers for renal dysfunction. In addition, hypercholesterolemia provoked alterations in glomerular size and structure. Similar results were reported in other studies, where experimentally induced hypercholesterolemia by HCD impaired renal functions and structure [38,39]. Likewise, in cardiac and hepatic tissues, hypercholesterolemic rats showed elevated oxidative stress and lipid peroxidation, which is evidenced by diminished GSH and antioxidant enzyme activities as well as elevated TBARS levels. Hypercholesterolemia is known to promote renal formation of free radicals leading to cellular injury and inflammation [40,41]. Ang II was found to endorse these effects in previous studies [42]. Furthermore, down-regulation of RAS activity by ACE inhibition or AT1R blocking showed attenuation of these renal impairments and oxidative stress as reported in the current and other studies [43,44].

The reported antioxidant effects of the AT1R blocker, LT, in the current study might be through a range of previously documented oxidative stress ameliorating processes. Studies showed that LT could inhibit nicotinamide adenine dinucleotide phosphate (NADPH) oxidase enzyme, which has an important role in the production of superoxide free radicals [45]. LT also might influence the production of NO *via* modulation of inducible NO synthases (iNOS) and endothelial NO synthases (eNOS) along with and NF-κB protein expression [46]. Evidences showed that losartan inhibits important pathways enrolled in the pathogenesis of inflammation and oxidative stress including ERK1/2 MAPK pathways [47]. In Wang et al. study, LT reduced the phosphorylation of p38, ERK, and p65, p50 nuclear transposition, which are important factors in MAPK and NF-κB pathways involved in several oxidative and inflammatory pathological conditions [48].

As a limitation to this study, the metabolic and oxidative alterations were measured only in male hypercholesterolemic rats, which may alter the assumption that animal gender and the associated hormonal differences may interfere with the therapeutic effects of RAS inhibitors. Another drawback of the current study is that food and water consumptions were not determined. Calculations of the food and water intake could have explained lipid profile variations between experimental groups.

## Conclusion

Taken together, this study confirmed the involvement of RAS in the metabolic impairments encountered following hypercholesterolemia caused by a cholesterol rich diet. Inhibition of the final step in the RAS process through blocking of AT1R by LT considerably protects against cardiac, hepatic, and renal metabolic abnormalities, oxidative injury, and inflammation in hypercholesterolemic animals. Therefore, the aforementioned beneficial effects of LT may promote it as a useful therapeutic tool to improve lipid profile and hypercholesterolemia-associated metabolic and oxidative alterations.

## Availability of data and materials

The datasets generated and/or analyzed in the present study are included in the manuscript.
